# Some Mental Symptoms Connected with the Catamenia

**Published:** 1883-02

**Authors:** 


### SOME MENTAL SYMPTOMS CONNECTED WITH THE CATAMENIA.

### Before Menses.

Anxiety: Carbo an., Cocc., Con., Merc., *Natr. m.*, Nit. ac., *Nux v.*, Stann., Sulph., Tarent.

Anxiety, with qualmishness: Natr. m.

Aversion to work: *Agar.*, Nux v.

Cheerful: Fluor. ac.

Excitable, very, day before: Mag. m.

Fearfulness: Carbo v., Natr. m., Sulph.

Ill-humor: Calc., Caust., *Cham.*, Kreos.

Irresistible, almost maniacal, desire for ardent spirits, has to get completely drunk, and feels afterward distressed—wants to be brought to an insane asylum: *Selen.*

Laughing: Hyos., Nux m.

Low-spirited, melancholic: Amm. c., Calc., *Caust.*, Cimic., Con., Cycl., Ferr., Lac. can., *Lyc.*, *Natr. m.*, Nit. ac., Phos., Plat., *Puls.*, Sil., *Stann.*, Vespa.

Madness, frenzy: *Acon.*, Bell., Hyos.

Nervous excitement: Crocus, Kreos., Mag. m., *Nux v.*

Restlessness: Acon., Coloc., Con., Kali c., Kreos., Mag. c., *Nux v.*, Sulph. (and anxiety day before).

SAD AND DOLEFUL THE DAY BEFORE: Nitr. ac.
SENSITIVE, she is very—throws herself on the bed and remains there all day: Sepia.
WEEPING: Cact. gr., Con., Lyc., Phos., Plat., *Puls.*

SYMPTOMS IN FULL.

ARS.: Neuralgic pain in teeth and head during frenzy—beats her head with fist.
CALC.: Timid and irritable day before.
CAUST.: Sees the dark side of everything.
CON.: Tearfulness, with heaviness of limbs—anxiety and restlessness. Anxiety about trifles.
HYOS.: Almost incessant loud laughter.
KREOS.: Irritability and restlessness some days before.
LYC.: Melancholy and out of humor. Raving and weeping, as if she were going mad, day and during first day.
MAG. M.: Irritable day before. Moves from side to side; cannot bear to be uncovered. Thick, black blood night before.
MANCIN.: Sadness, moaning, before menses.
NATR. M.: Anxiety. Anxious and faint in the morning, with sweet taste and a spitting of a little blood in the saliva.
NITR. AC.: Melancholy and anxious in the evening.
SEPIA: Very sensitive from a slight cause, with loss of appetite. Attack of despair. The night before, at midnight, chill, followed by heat, especially in face, with restlessness.
STANN.: Great anxiety for a week before, with depression, ceasing when the flow comes.
SULPH.: Moroseness and apprehension, with uterine pain. Two days too late, with anxious and uncomfortable feeling.

DURING MENSES.

ANXIETY: Aloe, Bell., Calc., Canth., Chel., Cimic., Con., Coff., Ign., Kali c., Kali hyd., Mag. c., Merc., Mosch., Natr. m., Nitr. ac., Phos., Rhod., Secale, Sil., Tarent., Zinc.
ANXIOUS RESTLESSNESS, followed by faintness: Calc.
AVERSION TO LABOR: Aloe, Rhod.
AVERSION TO LABOR, ESPECIALLY MENTAL: Agar.
CROSS, UNCIVIL, ETC., especially at the appearance of the menses: *Cham.*
DELIRIUM: Acon., Bell., Hyos., Lyc., Nux m.
— and rage: Acon., Hyos.
— — — wants to bite, to escape: Bell.
DESIRE TO BE ALONE: Cicuta, Elaps, Nux v.
DISINCLINATION TO ANSWER, second day: Zinc.
DULLNESS: Phos., Nux m.
FAULT FINDING: Nux v.
FEAR: Aloe.
— of being alone: Ars., Elaps.

FEAR OF DEATH: *Acon.*, Plat., Tarent.
— — every person; stares wildly about: Hyos.
FEARFULNESS: Mag. c., Phos.
— expecting something to creep out of every corner: Phos.
GRIEF: Graph., Pul.
— silent: Ign.
HURRIED, everything she does seems to be done too slowly: Ph. ac.
INDIFFERENCE: Rhod.
— even to those she loves: Merc., Sepia.
INTOLERANCE OF NOISE AND LIGHT: Ign., Nux m.
IRRITABLE, IMPATIENT, ILL-HUMOR: Amm. c., Ars., Berb., *Bry.*, Cact. g., Castor, Caust., *Cham.*, Cinnab., Cimic., Cop., Ferr., Ind., Kali c., Mag. c., Mag. s., *Nux v.*, Plat., Sulph., Tarent., Zinc., Zing.
LAMENTING, MOANING; exposes and uncovers herself: Hyos.
LAUGHING, convulsive: Cupr.
— excessive, tendency to: Nux m.
LOQUACIOUS: Lach., Stram.
— afterward stupid and irritable: Lachn.
— and bright: Glon.
— devout and beseeching: Stram.
— given to mirth: Crocus.
LOW-SPIRITED: *Amm. c.*, Berb., Brom., Cact. g., Calc., Caust., Chelid., *Cimic.*, Cop., Curare, Ferr., Graph., Ign., Ind., Lac. can., Lob., Mag. m., Mang., Mur. ac., Natr. c., *Natr. m.*, Petro., Plat., Plb., *Puls.*, Sepia, Sil., Zinc.
— on account of an indescribable, queer, ill feeling all over the body: Brom.
— very melancholy, with palpitation of heart and headache every morning on waking: Natr. m.
— with weeping: Ign., Plat., *Puls.*
MANIA FROM PROFUSE MENSES: *Sepia.*
MEMORY LOST: Amm. c., Lac. can., Zinc.
MOANING: Cicuta, Cocc., Coloc.
NERVOUS, cannot bear any noise: Natr. c.
— distress: Calc., Cocc.
— excitable: Cimic., Lach., Mag. m., Puls.
PEEVISH: Caust., Mag. c.
PROSTRATION AND MENTAL DEPRESSION: Ferr.
RESTLESSNESS: *Acon.*, Ars., Calc., Canth., Cham., Cimic., Coff., Coloc., Con., Gels., Ipec., Kali hyd., Plat., Rhus, Secale.
— at the beginning of the menses: Plat.
— followed by fainting: Calc.
RESTLESS, starts at least noise; unconquerable sadness during menses: Amm. c.
SENSITIVENESS, to external influences, noise, light, etc.: Asarum, *Nux v.*
SIGHING, SOBBING: Cimic., Cocc., Ign., Plat.

STUPOR: Nux m.
TALK, does not wish to, or to hear others: *Amm. c.*, Elaps.
TALK NOR THINK, does not wish to: Senecio.
THINKS SHE IS GOING TO DIE: Mosch.
THOUGHTS, HORRIFYING: Plat.
VIOLENT IN ALL ACTIONS: Cicuta.
WEARY OF LIFE: Aur., Berb., Merc., Sil.
WEEPING MOOD: Ars., Con., Graph., *Ign.*, Lach., Lyc., Plat., *Puls.*, *Lyc.*, Thuja, Zinc.
WEEPING AND RAVING FIRST DAY: Lyc.
— at the beginning of menses: Lyc., Puls.
— inclined to: Alum., Berb., Petrol.

SYMPTOMS IN FULL.

AMM. C.: Invincible sadness. Melancholy, with peevish fretfulness.
ARS.: Moans, lamentations, and tears with the gastric and abdominal pains.
BERB.: Ill humor and weariness of life, with pain of the vagina, anus, and upper extremities.
CALC: Tearful and confused.
CASTOR: Everything irritates; speaking is too much trouble.
CAUST.: Ill humor and tired feeling.
CHAM.: Quarrelsome and intolerant.
FERR.: Spiritless and exhausted after slight exertion.
KALI HYD.: Anxiety, with heat of head.
NATR. M.: Anxiety and faintness, with cold feeling in pelvis and inward heat.
NITR. AC.: Anxiety and heat for half an hour, with palpitation and tremor of limbs.
SILIC.: Melancholic anxiety—impelling her to drown herself.
SEPIA: Heaviness of the spirits in the morning. (Forenoon: Mag. c.)

### AFTER MENSES.

ANXIETY AND GREAT WEAKNESS: Phos.
— with interrupted sleep, at night: Agar.
EXHAUSTION OF MIND AND BODY, particularly after the least exertion: Alum.
GROANING: Stram.
ILL HUMOR: Berb., Bufo.
— and restlessness, with toothache: Mag. c.
LOW-SPIRITED: Alum., Ferr., Silic.
NERVOUS IRRITABILITY: Carb. ac.
WEEPING: Alum.
— whining, sobbing: Stram.

SYMPTOMS IN FULL.

ALUM.: Mental and bodily depression with despairing mood.
PALLAD: Feels sore in the abdomen after menses, with fear and apprehension that something horrible will happen.

